# Supplementary material for: Tract-specific statistics based on diffusion-weighted probabilistic tractography
Source: Commun Biol. 2022 Feb 17;5:138. doi: 10.1038/s42003-022-03073-w (PMC8854429; doi:10.1038/s42003-022-03073-w)
Supplement: Supplementary file 2 — Supplementary Information [file 42003_2022_3073_MOESM2_ESM.pdf]

Tract-specific statistics based on diffusion-weighted probabilistic tractography

Andrew T. Reid[1*], Julia A. Camilleri[2,3], Felix Hoffstaedter[2,3] and Simon B. Eickhoff[2,3]

[1*]School of Psychology, University of Nottingham, Nottingham, United Kingdom.
[2]Institute for Neuroscience and Medicine (INM-7), Jülich Research Center, Jülich, Germany.
[3]Institute of Systems Neuroscience, Medical Faculty, Heinrich Heine University, Düsseldorf, Germany.

*Corresponding author(s). E-mail(s): andrew.reid@nottingham.ac.uk;

1 Supplementary Material

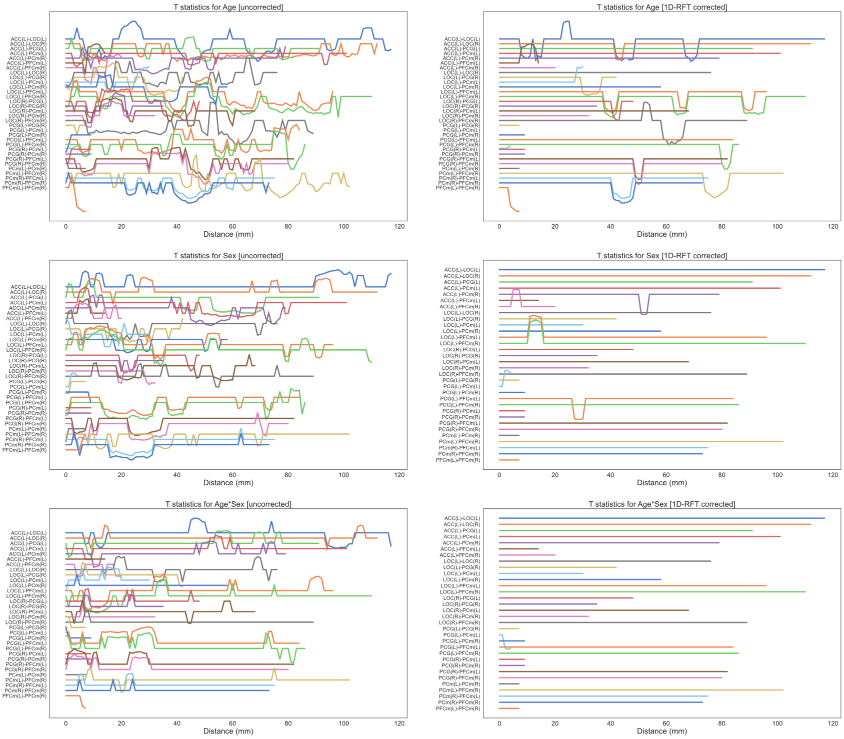

Supplementary Figure 1 GLM t-value distance traces for all ROI pairs in the DMN. Distance traces show the t-values for Age, Sex, and $Age \times Sex$, for all ROI pairs in the DMN. Plots on the left show uncorrected t-values, and plots on the right show t-values derived from one-dimensional random field theory (1D-RFT) and false-discovery rate (FDR) ¡ 0.05. Line separation is $t = 1$. N=130.

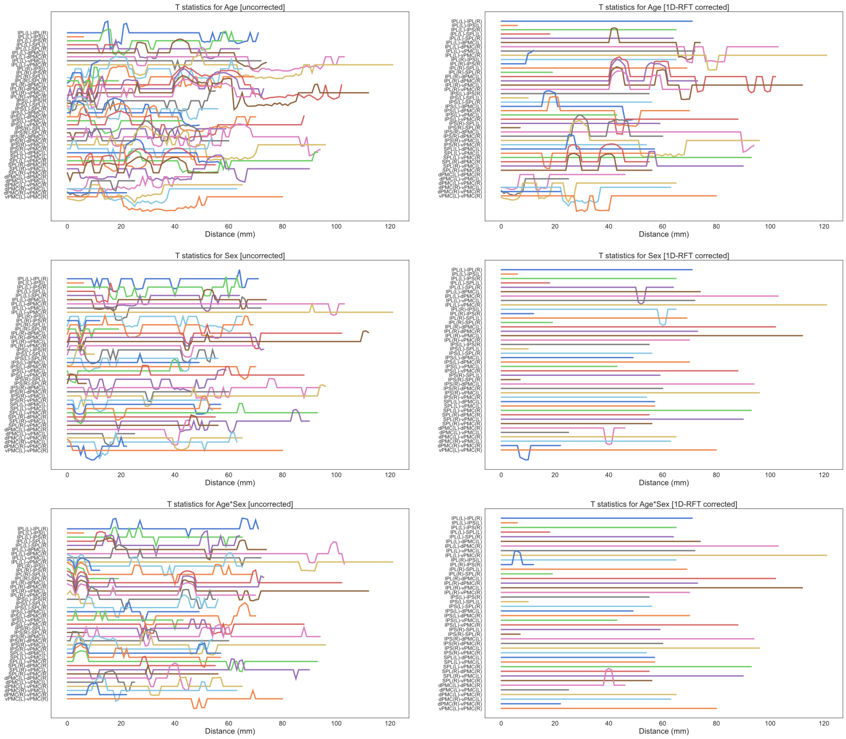

Supplementary Figure 2 GLM t-value distance traces for all ROI pairs in the WWN. Distance traces show the t-values for *Age*, *Sex*, and *Age* $\times$ *Sex*, for all ROI pairs in the WWN. Plots on the left show uncorrected t-values, and plots on the right show t-values derived from one-dimensional random field theory (1D-RFT) and false-discovery rate (FDR) ¡ 0.05. Line separation is $t = 1$. N=130.

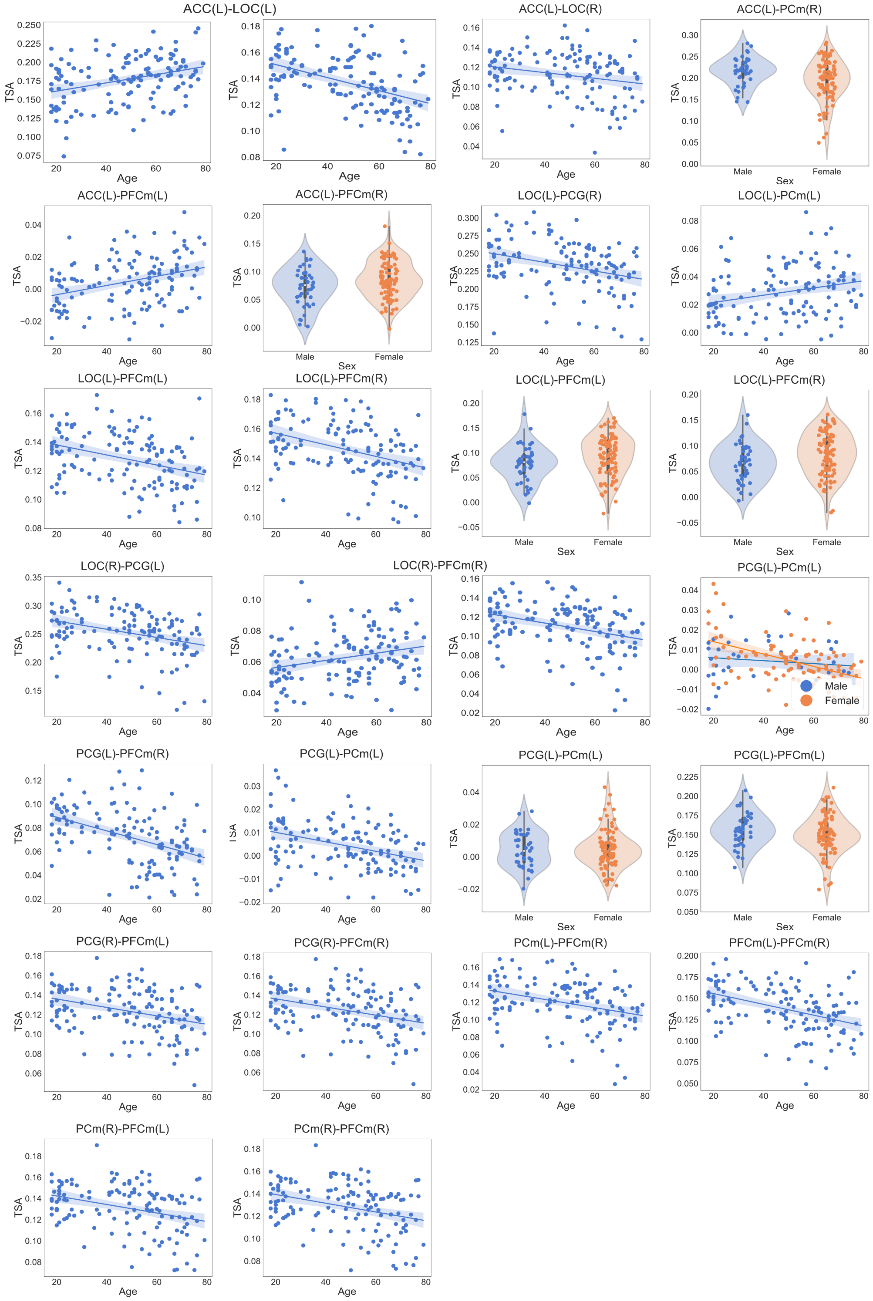

Supplementary Figure 3 Scatter and violin plots showing significant effects for all tracts in the DMN. N=130.

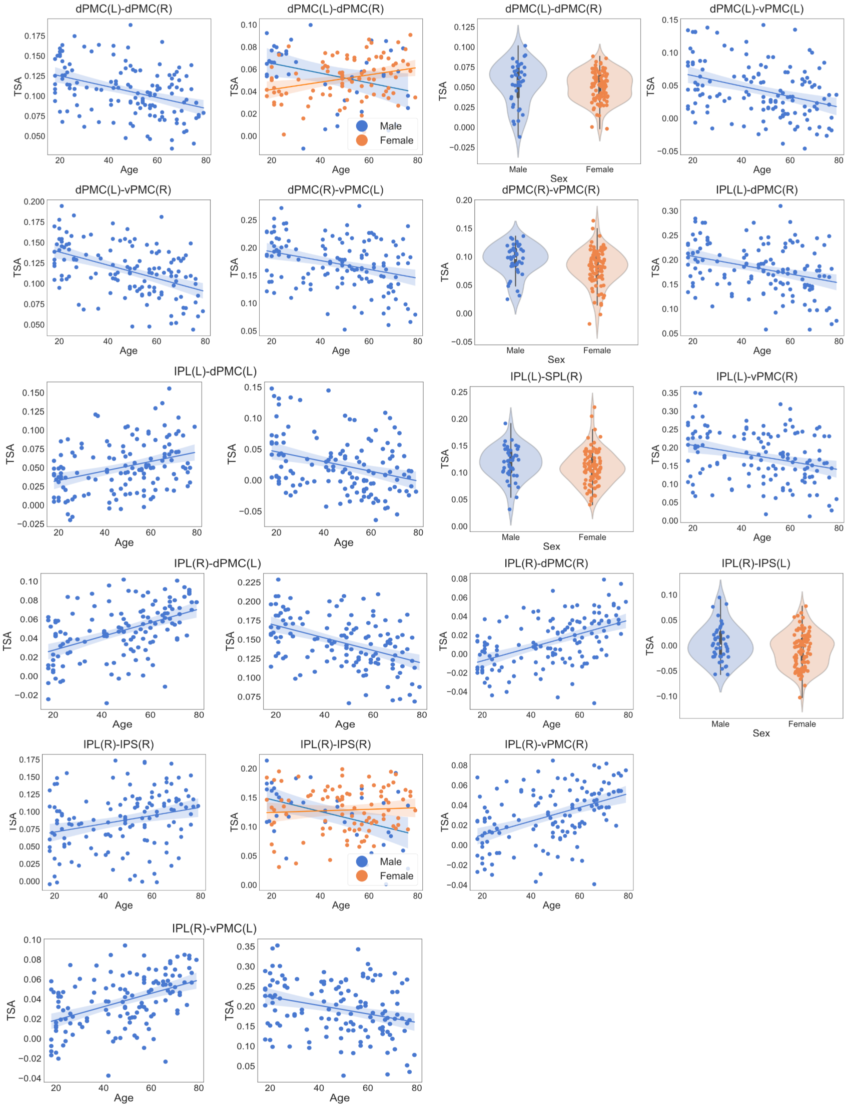

Supplementary Figure 4 Scatter and violin plots showing significant effects for all tracts in the WWN (Part A). N=130.

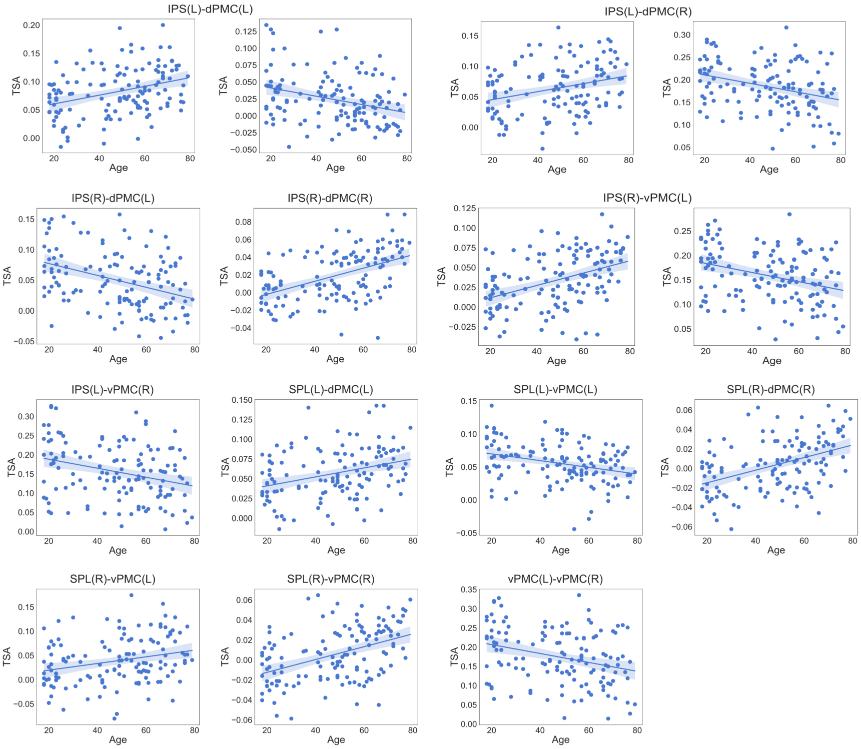

Supplementary Figure 5 Scatter and violin plots showing significant effects for all tracts in the WWN (Part B). N=130.

Supplementary Table 1 Statistics for all significant GLM effects in the DMN, sorted by effect size R^2 (N=130). Effect (+/-): whether the effect is positive or negative. $\sum t$: sum of t values in the cluster. R^2: proportion of explained variance for the full model, averaged across all cluster vertices.

**Factor**	**From**	**To**	**Effect**	$\sum$ **t**	$\mathbf{R^2}$
Age	PCG_L	PFCm_R	-	18.01	0.13
Age	PFCm_L	PFCm_R	-	18.15	0.13
Age	LOC_R	PFCm_R	-	34.25	0.12
Age	PCm_L	PFCm_R	-	38.14	0.12
Age	PCG_R	PFCm_L	-	12.05	0.11
Age	PCG_R	PFCm_R	-	11.99	0.11
Age	ACC_L	LOC_L	-	75.18	0.10
Age	PCm_R	PFCm_L	-	26.50	0.10
Age	PCm_R	PFCm_R	-	30.02	0.10
Age	LOC_R	PCG_L	-	11.14	0.09
Age	PCG_L	PCm_L	-	18.44	0.09
Age	ACC_L	PFCm_L	+	21.24	0.09
Age	LOC_L	PFCm_L	-	43.87	0.09
Age	LOC_L	PFCm_R	-	64.45	0.09
Age	LOC_R	PFCm_R	+	10.81	0.09
Sex	LOC_L	PFCm_R	+	17.84	0.09
Age	ACC_L	LOC_L	+	10.70	0.08
Age	ACC_L	LOC_R	-	9.93	0.08
Age	LOC_L	PCG_R	-	23.86	0.08
Sex	LOC_L	PFCm_L	+	17.00	0.08
Sex	PCG_L	PFCm_L	-	13.43	0.08
Sex	ACC_L	PCm_R	-	9.74	0.07
Age	LOC_L	PCm_L	+	9.18	0.07
Sex	ACC_L	PFCm_R	+	8.42	0.06
Sex	PCG_L	PCm_L	+	7.33	0.04
AgeXSex	PCG_L	PCm_L	-	6.71	0.03

Supplementary Table 2 Statistics for all significant GLM effects in the WWN, sorted by effect size R^2. Effect (+/-): whether the effect is positive or negative. $\sum t$: sum of t values in the cluster. R^2: proportion of explained variance for the full model, averaged across all cluster vertices.

Factor	From	To	Effect	$\sum$ t	$\mathbf{R^2}$
Age	IPS_R	dPMC_L	-	19.98	0.11
Age	IPS_R	vPMC_L	+	28.27	0.10
Age	IPL_R	dPMC_R	+	56.10	0.10
Age	IPS_R	dPMC_R	+	54.82	0.10
Age	IPL_R	dPMC_L	+	49.17	0.09
Age	SPL_R	dPMC_R	+	51.95	0.09
Age	IPL_R	vPMC_L	+	33.26	0.09
Age	IPL_L	dPMC_L	-	21.93	0.09
Age	SPL_R	vPMC_R	+	39.61	0.09
Sex	dPMC_L	dPMC_R	-	10.78	0.09
Age	dPMC_L	vPMC_R	-	60.61	0.09
Sex	IPL_L	SPL_R	-	10.65	0.09
Age	IPS_R	vPMC_L	-	59.69	0.09
Age	IPL_R	vPMC_R	+	39.59	0.08
Age	IPL_L	dPMC_R	-	38.31	0.08
Age	IPL_R	dPMC_L	-	31.09	0.08
Age	IPS_L	vPMC_R	-	10.39	0.08
Age	dPMC_R	vPMC_L	-	48.06	0.08
Age	IPS_L	dPMC_R	-	30.88	0.08
Age	IPS_L	dPMC_L	+	17.10	0.08
Sex	IPL_R	IPS_L	-	10.25	0.08
Age	vPMC_L	vPMC_R	-	37.60	0.08
Age	IPL_L	vPMC_R	-	13.67	0.08
Age	SPL_L	vPMC_L	-	10.21	0.08
Age	SPL_R	vPMC_L	+	10.24	0.08
Age	dPMC_L	vPMC_L	-	30.40	0.08
AgeXSex	dPMC_L	dPMC_R	+	10.15	0.08
Age	IPL_R	vPMC_L	-	13.31	0.08
Age	dPMC_L	dPMC_R	-	39.14	0.08
Age	IPL_L	dPMC_L	+	9.94	0.07
Age	SPL_L	dPMC_L	+	9.73	0.07
Age	IPS_L	dPMC_R	+	12.75	0.07
Age	IPS_L	dPMC_L	-	12.73	0.07
Sex	dPMC_R	vPMC_R	-	12.13	0.07
AgeXSex	IPL_R	IPS_R	+	8.33	0.06
Age	IPL_R	IPS_R	+	8.23	0.05

Supplementary Table 3 Typical processing times for a single subject on a CPU core (note that BedpostX is only run once).

**Network**	**Step**	**Processing time (hours)**
DMN	BedpostX	40.3
	ProbtrackX	75.0
	Tract determination	3.0
	TSA computation	0.2
	GLM analysis	1.1
	**Total**	**119.6**
WWN	BedpostX	40.3
	ProbtrackX	83.4
	Tract determination	3.2
	TSA computation	0.3
	GLM analysis	1.3
	**Total**	**128.5**
	**Total both**	**207.8**
