# Supplementary material for: Tract-specific statistics based on diffusion-weighted probabilistic tractography
Source: Commun Biol. 2022 Feb 17;5:138. doi: 10.1038/s42003-022-03073-w (PMC8854429; doi:10.1038/s42003-022-03073-w)
Supplement: Supplementary file 3 — Description of Additional Supplementary Files [file 42003_2022_3073_MOESM3_ESM.pdf]

Description of Additional Supplementary Files

File name: Supplementary Data 1
Description: All t-values trace data points for the DMN (Supplementary Figure 1). All data points used in the scatterplots and violin plots for ROI pairs in the DMN (Figure 6 and Supplementary Figure 3). Data is in CSV format.

File name: Supplementary Data 2
Description: All t-values trace data points for the WWN (Supplementary Figure 2). All data points used in the scatterplots and violin plots for ROI pairs in the WWN (Figure 6 and Supplementary Figures 4 and 5). Data is in CSV format.
