# Supplementary material for: Tract-specific statistics based on diffusion-weighted probabilistic tractography
Source: Commun Biol. 2022 Feb 17;5:138. doi: 10.1038/s42003-022-03073-w (PMC8854429; doi:10.1038/s42003-022-03073-w)
Supplement: Supplementary file 6 — Reporting Summary [file 42003_2022_3073_MOESM6_ESM.pdf]

nature research

Corresponding author(s): Andrew T. Reid

Last updated by author(s): Dec 12, 2021

# Reporting Summary

Nature Research wishes to improve the reproducibility of the work that we publish. This form provides structure for consistency and transparency in reporting. For further information on Nature Research policies, see our Editorial Policies and the Editorial Policy Checklist.

## Statistics

For all statistical analyses, confirm that the following items are present in the figure legend, table legend, main text, or Methods section.

| n/a | Confirmed | |
|---|---|---|
| ☐ | ☒ | The exact sample size (*n*) for each experimental group/condition, given as a discrete number and unit of measurement |
| ☒ | ☐ | A statement on whether measurements were taken from distinct samples or whether the same sample was measured repeatedly |
| ☐ | ☒ | The statistical test(s) used AND whether they are one- or two-sided<br>*Only common tests should be described solely by name; describe more complex techniques in the Methods section.* |
| ☐ | ☒ | A description of all covariates tested |
| ☐ | ☒ | A description of any assumptions or corrections, such as tests of normality and adjustment for multiple comparisons |
| ☐ | ☒ | A full description of the statistical parameters including central tendency (e.g. means) or other basic estimates (e.g. regression coefficient) AND variation (e.g. standard deviation) or associated estimates of uncertainty (e.g. confidence intervals) |
| ☐ | ☒ | For null hypothesis testing, the test statistic (e.g. *F*, *t*, *r*) with confidence intervals, effect sizes, degrees of freedom and *P* value noted<br>*Give P values as exact values whenever suitable.* |
| ☒ | ☐ | For Bayesian analysis, information on the choice of priors and Markov chain Monte Carlo settings |
| ☒ | ☐ | For hierarchical and complex designs, identification of the appropriate level for tests and full reporting of outcomes |
| ☐ | ☒ | Estimates of effect sizes (e.g. Cohen's *d*, Pearson's *r*), indicating how they were calculated |

*Our web collection on statistics for biologists contains articles on many of the points above.*

## Software and code

Policy information about availability of computer code

| | |
|---|---|
| Data collection | No data were collected for this study; the data are from a publicly available neuroimaging data set. |
| Data analysis | FSL version 6.0.0 software was used for preprocessing, probabilistic tractography, and spatial transformation of imaging data. Custom Python software was used to perform the main processing and statistical analyses, based on several standard Python dependencies; in particular, \| nibabel was used for image operations, and statsmodels was used for linear regression analysis and FDR correction, rft1d was used for random field inference, and seaborn was used for plotting. |

For manuscripts utilizing custom algorithms or software that are central to the research but not yet described in published literature, software must be made available to editors and reviewers. We strongly encourage code deposition in a community repository (e.g. GitHub). See the Nature Research guidelines for submitting code & software for further information.

## Data

Policy information about availability of data

All manuscripts must include a data availability statement. This statement should provide the following information, where applicable:

- Accession codes, unique identifiers, or web links for publicly available datasets
- A list of figures that have associated raw data
- A description of any restrictions on data availability

The Enhanced NKI-Rockland dataset used in this study is freely available at www.nitrc.org/projects/fcon_4621000/. Derived participant- and sample-wise data, along with parameter files specifying the processing and analyses steps used to generate them, are freely available from the University of Nottingham Research Data Management Repository at https://doi.org/10.17639/nott.7102.

# Field-specific reporting

Please select the one below that is the best fit for your research. If you are not sure, read the appropriate sections before making your selection.

[x] Life sciences  [ ] Behavioural & social sciences  [ ] Ecological, evolutionary & environmental sciences

For a reference copy of the document with all sections, see nature.com/documents/nr-reporting-summary-flat.pdf

# Life sciences study design

All studies must disclose on these points even when the disclosure is negative.

| | |
|---|---|
| Sample size | Sample size was based on available data from the Enhanced NKI Rockland data set. |
| Data exclusions | Participants with clinical diagnoses were excluded on the basis that they likely deviated from the "normal" population used to generate average tract trajectory estimates. |
| Replication | Findings were not replicated, but replication with additional data sets is possible via the provided software. |
| Randomization | This is not relevant (no experimental groups/conditions were defined). |
| Blinding | This is not relevant (no experimental groups/conditions were defined). |

# Reporting for specific materials, systems and methods

We require information from authors about some types of materials, experimental systems and methods used in many studies. Here, indicate whether each material, system or method listed is relevant to your study. If you are not sure if a list item applies to your research, read the appropriate section before selecting a response.

## Materials & experimental systems

| n/a | Involved in the study | |
|---|---|---|
| [x] | [ ] | Antibodies |
| [x] | [ ] | Eukaryotic cell lines |
| [x] | [ ] | Palaeontology and archaeology |
| [x] | [ ] | Animals and other organisms |
| [ ] | [x] | Human research participants |
| [x] | [ ] | Clinical data |
| [x] | [ ] | Dual use research of concern |

## Methods

| n/a | Involved in the study | |
|---|---|---|
| [x] | [ ] | ChIP-seq |
| [x] | [ ] | Flow cytometry |
| [ ] | [x] | MRI-based neuroimaging |

## Human research participants

Policy information about studies involving human research participants

| | |
|---|---|
| Population characteristics | 130 participants (86 female, age range 18 to 80) were obtained from the Enhanced NKI Rockland sample. Participants were recruited in Rockland County, NY, with an ethnic composition that largely reflected that of the United States (see Nooner et al., 2012, Table 1). |
| Recruitment | N/A - we did not recruit |
| Ethics oversight | N/A - we did not recruit |

Note that full information on the approval of the study protocol must also be provided in the manuscript.

## Magnetic resonance imaging

### Experimental design

| | |
|---|---|
| Design type | Only anatomical scans were used. |
| Design specifications | N/A |
| Behavioral performance measures | N/A |

## Acquisition

| | |
|---|---|
| Imaging type(s) | MP-RAGE, DWI |
| Field strength | 3.0T |
| Sequence & imaging parameters | MP-RAGE (TR = 1900 ms; TE = 2.52 ms, 1mm isotropic); DWI (TR = 2400 ms; TE = 85 ms; voxel size = 2 mm isotropic; b = 1500 s/mm2; 137 gradient directions) |
| Area of acquisition | Whole brain |

Diffusion MRI ☒ Used ☐ Not used

Parameters: TR = 2400 ms; TE = 85 ms; voxel size = 2 mm isotropic; b = 1500 s/mm2; 137 gradient directions; multiband factor: 4

## Preprocessing

| | |
|---|---|
| Preprocessing software | FSL 6.0.0; brain extraction with BET; eddy correction with EDDY |
| Normalization | Linear and nonlinear normalization was performed on diffusion FA images using FLIRT and FNIRT. |
| Normalization template | FA template, specified in MNI-152 space |
| Noise and artifact removal | *Describe your procedure(s) for artifact and structured noise removal, specifying motion parameters, tissue signals and physiological signals (heart rate, respiration).* |
| Volume censoring | *Define your software and/or method and criteria for volume censoring, and state the extent of such censoring.* |

## Statistical modeling & inference

| | |
|---|---|
| Model type and settings | Mass-univariate general linear models with first order and interaction terms |
| Effect(s) tested | F test of full model: TSA ~ Age + Sex + Age X Sex, t-tests of coefficients for each term |

Specify type of analysis: ☐ Whole brain ☒ ROI-based ☐ Both

Anatomical location(s): Tract trajectory estimates were determined using the method described in the article

| | |
|---|---|
| Statistic type for inference (See Eklund et al. 2016) | One-dimensional random field theory cluster-wise inference on t statistics projected onto the core tract trajectory |
| Correction | FDR (q<0.05) was performed over all clusters in all tracts simultaneously. |

## Models & analysis

| n/a | Involved in the study |
|---|---|
| ☒ | ☐ Functional and/or effective connectivity |
| ☒ | ☐ Graph analysis |
| ☒ | ☐ Multivariate modeling or predictive analysis |
